# Examination of the regression model to quantify the degree of low back pain and lower limb symptoms in patients with lumbar disc herniation by the Japanese Orthopaedic Association Back Pain Evaluation Questionnaire (JOABPEQ)

**DOI:** 10.1371/journal.pone.0243861

**Published:** 2020-12-14

**Authors:** Hayato Ishitani, Toshiyo Tamura, Shigehiko Kanaya, Hiroshi Fujimoto

**Affiliations:** 1 Department of Rehabilitation, Funabashi Orthopedic Hospital Nishifuna Clinic, Funabashi City, Chiba Prefecture, Japan; 2 Institute of Healthcare Robotics, Future Robotics Organization, Waseda University, Shinjuku-ku, Tokyo, Japan; 3 Computational Systems Biology Laboratory, Nara Institute of Science and Technology, Ikoma City, Nara Prefecture, Japan; University of California Berkeley, UNITED STATES

## Abstract

The Japanese Orthopedic Association Back Pain Evaluation Questionnaire (JOABPEQ) was created to evaluate specific treatment outcomes in terms of physical functioning, social ability, and mental health in patients with back pain-related diseases. In this study, we investigated whether the JOABPEQ could be used to construct a regression model to quantify low back pain and lower limb symptoms in patients with lumbar disc herniation (LDH). We reviewed 114 patients with LDH scheduled to undergo surgery at our hospital. We measured the degrees of 1) lower back pain, 2) lower limb pain, and 3) lower limb numbness using the visual analog scale before the surgery. All answers and physical function data were subjected to partial least squares regression analysis. The degrees of lower back and lower limb pain could be used as a regression model from the JOABPEQ and had a significant causal relationship with them. However, the degree of lower limb numbness could not be used for the same. Based on our results, the questions of the JOABPEQ can be used to multilaterally understand the degree of lower back pain and lower limb pain in patients with LDH. However, the degree of lower limb numbness has no causal relationship, so actual measurement is essential.

## Introduction

Lower back pain leads to physical function and pathology problems and impairs activities of daily living (ADL) and causes psychological problems [1]. Also, given that patients' problems are multifaceted, it is necessary to evaluate treatment results from multiple perspectives.

In recent years, the importance of evidence-based medicine has been emphasized, and various evaluation criteria require quantitative data regarding physical functions. It has been reported that low back pain treatment requires physiotherapy to improve function and willingness and adaptability to fight the disease on the part of patients and empathize on the part of the physician [2, 3]. Lower back pain is not a sensation that can be physically communicated, and it is difficult to quantify objectively. Pain sensitivity depends on individual pain experience. Also, differences in pain thresholds and pain expression arise from differences in age, gender, and cultural background. The pain threshold also varies between individuals from similar backgrounds, depending on physical, psychological, and social situations.

During clinical practice, we often observe different degrees of pain reported by patients. Some patients may be highly tolerant to pain, even though their conditions are so painful that they cannot walk comfortably, whereas others may not be so tolerant. Thus, pain assessment depends on the patient's subjective pain complaints, regardless of physical function.

The Japanese Society of Orthopedic Surgery created the Japan Orthopedic Association Low Back Pain Assessment Questionnaire (JOABPEQ) in 2007 from this perspective.

The JOABPEQ combines the low back pain-specific quality of life (QOL) scale of the Roland–Morris disability questionnaire (RDQ) and the comprehensive health index of the Short-Form 36 Health Survey (SF-36) [1], reduced from 60 to 25 items. There are five question domains: pain-related disorders, lumbar dysfunction, gait dysfunction, social life disorders, and psychological disorders.

Each JOABPEQ domain is calculated with a value range of 0–100 points, with higher values being better [1]. The JOABPEQ is a patient-based evaluation method that does not consider any therapist bias; it assesses QOL specifically concerning back pain. As an added benefit, it is an evaluation standard that can be understood not only by healthcare professionals but also by third parties. It is useful as a method of investigating pain levels before and after surgery and other interventions to determine therapeutic effects. The JOABPEQ has been translated to Korean, Thai, Portuguese (Brazil), and Persian (Iran) [4]. This translation was possible because of the simplicity and shortened length of the questionnaire.

Previous studies on the JOABPEQ used it before and after the intervention to evaluate the effects of treatments such as lumbar spine surgery and physical therapy. A study using correlation analysis reported that the degree of back pain correlates with the pain, walking function, and social life domains of the JOABPEQ. Therefore, studies using the JOABPEQ focus primarily on comparative testing and correlation analysis for the degree of back pain [5, 6]. However, no study has investigated whether the 25 items of the JOABPEQ can explain the degree of lower limb symptoms in patients with low back pain-related diseases. We performed a multivariate linear regression analysis to determine whether the JOABPEQ could address low back pain and lower limb symptoms. We also examined whether a regression model showing the degree of low back pain and lower limb symptoms could be constructed from the answers from the JOABPEQ. If the degree of subjective low back pain and lower limb symptoms complained about by the patient can be quantified from questions such as lumbar spine function, gait function, and ADL in the JOABPEQ, we can provide stepwise physical therapy according to the patient's pain condition.

The study analyzed the data of 114 patients with lumbar disc herniation (LDH) scheduled for surgery at Funabashi Orthopedic Hospital. Then, we collected data on their answers to the JOABPEQ and lower back pain and lower limb symptoms and identified questions that could significantly affect the degree of lower back pain and lower limb symptoms. In this study, as a retrospective study, we investigated whether lower limb symptoms in patients with LDH scheduled for surgery at Funabashi Orthopedic Hospital correlated with the JOABPEQ responses. We then examined whether a regression model showing the degree of low back pain and lower limb symptoms could be constructed from the answers from the JOABPEQ in patients with LDH.

## Materials and methods

### Subjects

Between August 2015 and August 2017, 213 patients diagnosed with LDH at the Funabashi Orthopedic Hospital were included in the study. They did not see any improvement in their symptoms with conservative therapy and planned to undergo excision. Patients with lumbar spinal stenosis, lumbar spondylolisthesis, lumbar scoliosis, or a history of lumbar spine surgery were excluded. The JOABPEQ was distributed and collected, and 99 patients who had one or more blanks in the JOABPEQ questionnaire were excluded from the study. The remaining 114 patients (88 male, 26 female, average age of 50.2 ± 18.7 years) were analyzed.

**Fig 1:**
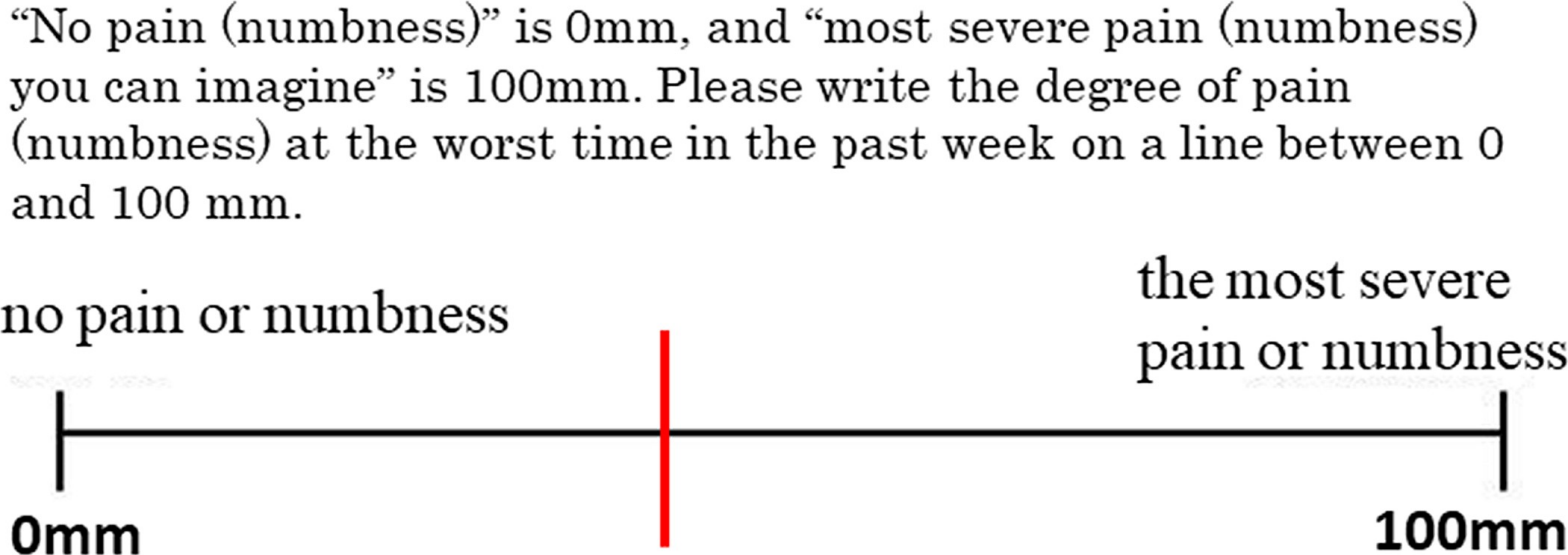
Visual analog scale; VAS. Assessment of the degree of low back pain, limb pain, and limb numbness.

Two trained physiotherapists performed the measurements. For pain evaluation, the degrees of lower back pain, lower limb pain, and lower limb numbness were measured using the visual analog scale (VAS) (Fig 1). For each parameter, the worst degree of symptoms in the previous week was pointed out on a line extending from 0 mm (no pain at all) to 100 mm (most painful to date).

All subjects consented and signed oral and written explanations of the purpose, method, and result's handling in this study. This study was also performed with the approval of the Ethics Committee of Funabashi Orthopedic Hospital (approval number: 2018013).

### Statistical analysis

In this study, partial least squares (PLS) regression analysis [7] was performed with objective variables as the degrees of lower back pain, lower limb pain, and lower limb numbness and the explanatory variables as answers to the JOABPEQ. The average value, predictive explanatory variance (Q^2^ value), and correlation coefficient (R-value) between the predicted value and the measured value were calculated by PLS regression analysis. Also, the objective variables with predictive relevance were examined using the coefficients of each item of the JOABPEQ. The closer the Q^2^ value is to one, the higher the predictability of the model. The correlation coefficient (R) is a real value between -1 and +1. A positive R-value indicates a positive correlation, a negative R-value indicates a negative correlation, and a zero R-value indicates no correlation. Answers to the JOABPEQ questions were digitized using an ordinal scale. The Q^2^ value is defined by **Formula 1**. Also, “y_obs_” and “y_calc_” indicate the PLS's measured values and calculated values, respectively.

Formula 1:

$$Q^{2}=1-\frac{\Sigma {\left(y_{obs}-y_{calc}\right)}^{2}}{\Sigma {\left(y_{obs}-\bar{y}\right)}^{2}}$$ (1)

Chin et al. [8] reported that the coefficient of determination of the predictive relevance was set to a Q^2^ value of zero or more. Regarding the objective variables with causal relationships, we focused on the absolute value of each question's coefficient in the JOABPEQ and examined the interrelationship with the objective variables. For statistical analysis, R (version 3.6.2) [9] was used.

The PLS method is a multiple regression analysis using the least-squares method between the principal components calculated from the explanatory variables and the objective variable. The effectiveness of its use in healthcare data analysis has been demonstrated [7]. A simple regression model can be constructed because it uses only partial information in the explanatory variables. Typically, regression analysis with a high correlation between two explanatory variables leads to overfitting, making the model unstable. However, the PLS method stabilizes the regression coefficient by making each variable uncorrelated and using only principal components smaller in number than the explanatory variables. Thus, the model's overfitting is less likely to occur, and the estimation error for new samples is reduced. Since the PLS method analyzes only the explanatory variables' partial information, a simple regression model can be created. Therefore, the PLS method was applied to this research method because it has the characteristic that a regression model can be created even if the number of explanatory variables is larger than the number of samples. Using the 25 explanatory variables (X1, X2, …, X25), the objective variable Y, which is the degree of lower back pain, the degree of lower limb pain, and the degree of lower limb numbness, is explained by **Formula 2** of the linear model. The 114 subjects were patients who visited the Funabashi Orthopedic Hospital and did not select particular subjects.

Formula 2:

$$Y=b_{0}+b_{1}X_{1}+b_{2}X_{2}+\ldots +b_{25}X_{25}$$ (2)

## Results

In this study, the average value of each domain of the JOABPEQ was 31.8 points for pain-related disorders, 44.4 points for lumbar dysfunction, 35.1 points for gait dysfunction, 31.2 points for social life disorders, and 42.9 points for psychological disorders.

Table 1 shows the mean (mm), predictive variance (Q^2^ value), and correlation coefficient (R-value), as determined by the PLS regression analysis.

**Table 1:** Result of the PLS regression analysis.

	Mean (mm)	Q^2^	R
1) Degree of lower back pain	54.60±31.3	0.144	0.292
2) Degree of lower limb pain	66.75±26.6	0.256	0.358
3) Degree of lower limb numbness	59.53±31.2	-0.032	0.189

According to the results of the PLS regression analysis, the degree of lower back pain had an average value of 54.60 mm, Q^2^ = 0.144, R = 0.292; the degree of lower limb pain had an average value of 66.75 mm, Q^2^ = 0.256, R = 0.358; and the degree of lower limb numbness had an average value of 59.53 mm, Q^2^ = -0.032, and R = 0.189.

The coefficient values of each item of the JOABPEQ in each objective variable are described below.

### Degrees of lower back pain

**Fig 2:**
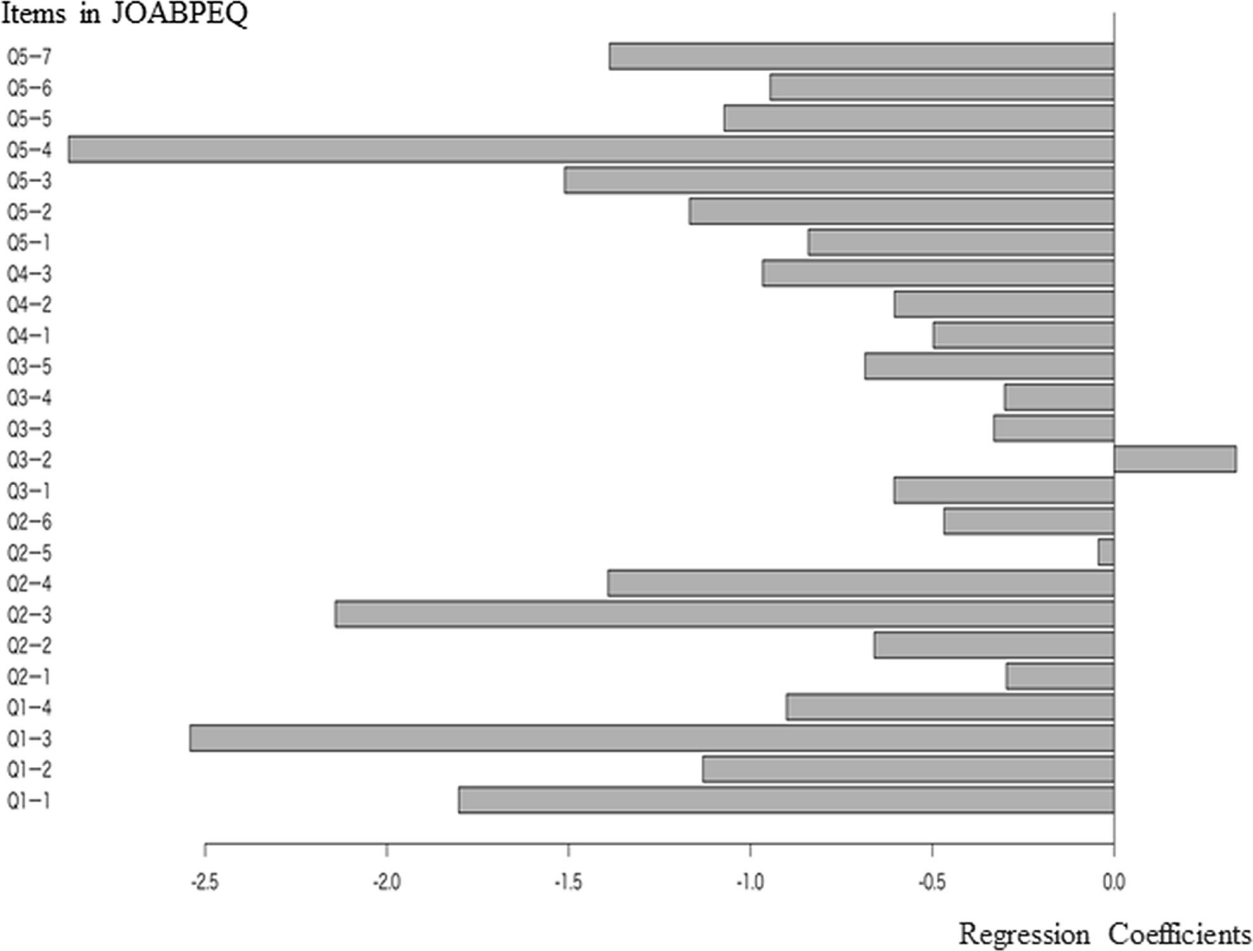
Regression coefficients of each JOABPEQ item for the degree of lower back pain.

The degree of lower back pain could be used as a regression model from the answers to the JOABPEQ and had a significant causal relationship with them (Q2 = 0.144). The correlation coefficient between the predicted and measured values showed a weak positive correlation (R = 0.292). Concerning the coefficient of each item of the JOABPEQ, those of pain-related, lumbar function, health, and psychological disorder were high (Fig 2).

The items with high absolute values of the coefficients shown in Table 2 show that the coefficients were higher for Q5-4 (Do you feel exhausted?), Q1-3 (Your lower back is almost always aching), Q2-3 (Because of low back pain, you have difficulty in standing up from a chair), Q1-1 (To alleviate low back pain, you often change your posture), Q5-3 (Have you been discouraged and depressed?), Q2-4 (Because of the low back pain, turning over in bed is difficult), Q5-7 (Do you feel your health will get worse?), Q5-2 (How is your present health condition?), Q1-2 (Because of low back pain, you lie down more often than usual), and Q5-5 (Have you felt happy?).

**Table 2:** Regression coefficients of each JOABPEQ item for the degree of lower back pain (in descending order of absolute value).

JOABPEQ	Coefficient	Absolute value	Contents of question
Q5-4	-2.88	2.87	Do you feel exhausted?
Q1-3	-2.54	2.54	Your lower back is almost always aching.
Q2-3	-2.14	2.14	Because of the low back pain, you have difficulty standing up from a chair.
Q1-1	-1.80	1.80	To alleviate low back pain, you often change your posture.
Q5-3	-1.51	1.51	Have you been discouraged and depressed?
Q2-4	-1.39	1.39	Because of the low back pain, turning over in bed is difficult.
Q5-7	-1.39	1.38	Do you feel your health will get worse?
Q5-2	-1.17	1.16	How is your present health condition?
Q1-2	-1.13	1.13	Because of the low back pain, you lie down more often than usual.
Q5-5	-1.07	1.07	Have you felt happy?
Q4-3	-0.97	0.96	Has your work routine been hindered because of the pain?
Q5-6	-0.95	0.94	Do you think you are in decent health?
Q1-4	-0.90	0.90	Because of the low back pain, you cannot sleep well. (If you take sleeping pills because of the pain, select “No.”)
Q5-1	-0.84	0.84	Because of the low back pain, you get irritated or get angry at other persons more often than usual.
Q3-5	-0.69	0.68	Do you have difficulty walking for more than 15 minutes?
Q2-2	-0.66	0.65	Because of the low back pain, you refrain from bending forward or kneeling down.
Q3-1	-0.61	0.60	Because of the low back pain, you walk only short distances.
Q4-2	-0.60	0.60	Have you been unable to do your work or ordinary activities as well as you would like?
Q4-1	-0.50	0.49	Because of the low back pain, you do not do any routine housework these days.
Q2-6	-0.47	0.46	Do you have difficulty in any one of the following motions; bending forward, kneeling, or stooping?
Q3-2	0.34	0.33	Because of the low back pain, you stay seated most of the day.
Q3-3	-0.33	0.33	Because of the low back pain, you go up the stairs more slowly than usual.
Q3-4	-0.30	0.30	Do you have difficulty in going up the stairs?
Q2-1	-0.30	0.29	Because of the low back pain, sometimes ask someone to help you when you do something.
Q2-5	-0.04	0.04	Because of the low back pain, you have difficulty putting on socks or stockings.

### Degrees of lower limb pain

**Fig 3:**
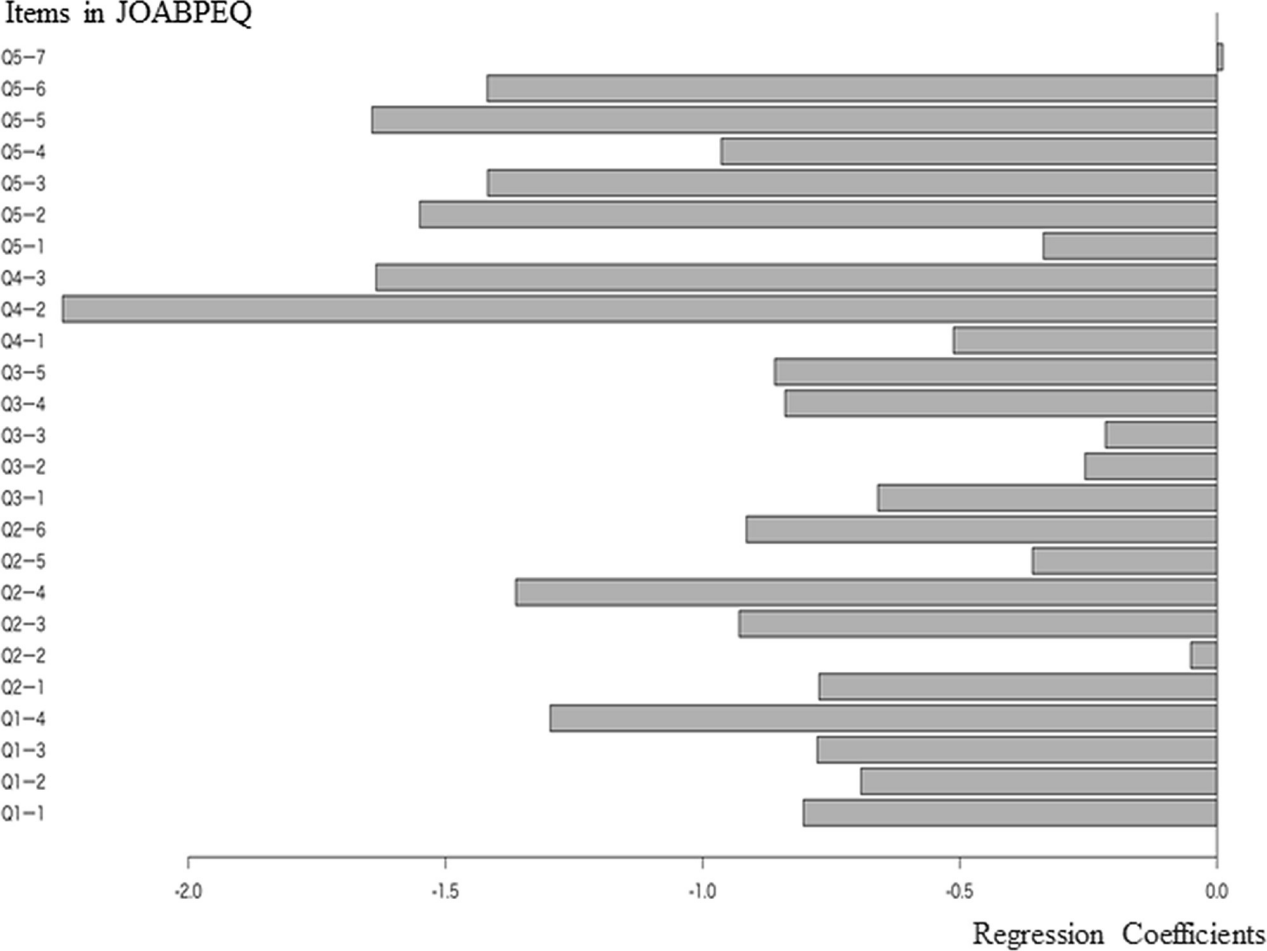
Regression coefficients of each JOABPEQ item for the degree of lower limb pain.

The degree of lower limb pain could be used as a regression model from the answers to the JOABPEQ and had a significant causal relationship with them (Q^2^ = 0.256). The correlation coefficient between the predicted and measured values showed a weak positive correlation (R = 0.358). Regarding the coefficient of each item of the JOABPEQ, those of social life, health, and psychological disorder were high (Fig 3).

The question items shown in Table 3 with high absolute values of the coefficients show that the coefficients were higher for Q4-2 (Have you been unable to do your work or ordinary activities as well as you would like?), Q4-3 (Has your work routine been hindered because of the pain?), Q5-5 (Have you felt happy?), Q5-2 (How is your present health condition?), Q5-3 (Have you been discouraged and depressed?), Q5-6 (Do you think you are in decent health?), Q2-4 (Because of low back pain, turning over in bed is difficult), and Q1-4 (Because of low back pain, you cannot sleep well).

**Table 3:** Regression coefficients of each JOABPEQ item for the degree of lower limb pain (in descending order of absolute value).

JOABPEQ	Coefficient	Absolute value	Contents of question
Q4-2	-2.24	2.24	Have you been unable to do your work or ordinary activities as well as you would like?
Q4-3	-1.64	1.64	Has your work routine been hindered because of the pain?
Q5-5	-1.64	1.64	Have you felt happy?
Q5-2	-1.55	1.55	How is your present health condition?
Q5-3	-1.42	1.42	Have you been discouraged and depressed?
Q5-6	-1.42	1.42	Do you think you are in decent health?
Q2-4	-1.36	1.36	Because of the low back pain, turning over in bed is difficult.
Q1-4	-1.29	1.29	Because of the low back pain, you cannot sleep well. (If you take sleeping pills because of the pain, select “No.”)
Q5-4	-0.96	0.96	Do you feel exhausted?
Q2-3	-0.93	0.93	Because of the low back pain, you have difficulty standing up from a chair.
Q2-6	-0.91	0.91	Do you have difficulty in any one of the following motions; bending forward, kneeling, or stooping?
Q3-5	-0.86	0.86	Do you have difficulty walking for more than 15 minutes?
Q3-4	-0.84	0.84	Do you have difficulty in going up the stairs?
Q1-1	-0.80	0.80	To alleviate low back pain, you often change your posture.
Q2-1	-0.78	0.78	Because of the low back pain, sometimes ask someone to help you when you do something.
Q1-3	-0.77	0.77	Your lower back is almost always aching.
Q1-2	-0.69	0.69	Because of the low back pain, you lie down more often than usual.
Q3-1	-0.66	0.66	Because of the low back pain, you walk only short distances.
Q4-1	-0.51	0.51	Because of the low back pain, you do not do any routine housework these days.
Q2-5	-0.36	0.36	Because of the low back pain, you have difficulty putting on socks or stockings.
Q5-1	-0.34	0.34	Because of the low back pain, you get irritated or get angry at other persons more often than usual.
Q3-2	-0.26	0.26	Because of the low back pain, you stay seated most of the day.
Q3-3	-0.22	0.22	Because of the low back pain, you go up the stairs more slowly than usual.
Q2-2	-0.05	0.05	Because of the low back pain, you refrain from bending forward or kneeling down.
Q5-7	0.01	0.01	Do you feel your health will get worse?

### Degrees of lower limb numbness

The degree of lower limb numbness could not be used as a regression model from the answers to the JOABPEQ and did not have a causal relationship with them (Q^2^ = -0.032).

## Discussion

The JOABPEQ had four items that could be used as indicators of lower back pain, but only some items were in the top rank, and most of the items related to social life, health condition, and psychological aspect. The JOABPEQ scores reflect independent questionnaires for each symptom. Correlations between items are an essential determinant of the mode of treatment by physiotherapists. The multiple regression model used an independent variable to predict the outcome. While the JOABPEQ could not predict psychological or social factors that were mainly responsible for the cause of pain, we were able to infer the degrees of lower back pain and lower leg pain from the JOABPEQ based on the PLS regression analysis of this study.

LDH is caused by mechanical stress arising from daily postures and movements of the intervertebral disc and degeneration due to aging. Symptoms of LDH include severe lower back pain, lower limb pain, and lower limb numbness. One of our aims was to make the JOABPEQ suitable for evaluating pain in adult LDH.

The VAS is a unidimensional, validated, subjective measure for acute and chronic pain intensity. It is widely used for evaluating pain because of its simplicity and adaptability to a broad range of populations and settings. If we can predict the degree of pain in patients from the JOABPEQ, the effectiveness of the JOABPEQ can be proven. The JOABPEQ can also help provide advice and exercise guidance before surgery in daily life based on the condition. The VAS was used to assess leg dysfunction, degree of leg numbness, and degree of low back pain. The VAS evaluates the intensity of pain, whereas the JOABPEQ presents the overall state of the health of patients with the lumbar disease. The PLS regression analysis showed a significantly positive correlation between the JOABPEQ and degrees of lower back and lower limb pain but a less significant correlation between the JOABPEQ and degree of lower limb numbness. The highest Q2 was obtained for the degree of leg pain. These results show that the JOABPEQ can predict degrees of lower back pain as well as lower limb pain.

Additionally, the variables of the degrees of lower back pain and lower limb pain were different. The degree of lower back pain mainly constructed a regression model based on pain, lumbar dysfunction, and psychological disorders, whereas lower limb pain mainly focused on psychological and social functions. As indicated earlier, mechanical and soft-tissue injuries are the most common causes of lower back pain. The lower back strain develops slowly over time from repetitive movements. Lower limb pain is typically worse than lower back pain. If the pain radiates along the path of the large sciatic nerve in the back of the leg, it is referred to as sciatica or radiculopathy. Lower limb pain can be intermittent or constant and can range from a dull ache to a searing, throbbing, or burning sensation. LDH patients have limited physical activities (physical components), social activities, and well-being (mental health component) owing to pain in the lower back and leg. Numbness may be experienced as a loss of sensation or a cold, icy feeling in one or more leg areas. Because there are several reasons for numbness, the predictor contains similar variants to obtain the coefficients.

Furthermore, Affleck et al. [10] stated that psychological factors significantly affect pain exacerbation and remission and that cognitive distortions such as reduced self-efficacy promote negative emotional rebound and physical reactions. The questionnaire items with high coefficients that influenced the prediction of lower back pain and lower limb pain were related to the health condition, psychological aspects, and ADL of rolling over and standing up. The previous evaluation used several health-related QOL scales such as the VAS and Numerical Rating Scale (NRS) and a questionnaire about subjective prognoses such as the RDQ, SF-36, and JOABPEQ. A predictive model was created from these scales and questionnaires; most studies presented a comparison between pre- and post-surgery. Mannion et al. [11] examined a predictive model of the overall score, including pain and function, ADL, and QOL, after spinal surgery, using multiple regression analysis. The preoperative psychosocial condition had the highest predictive coefficient. Block et al. [12] reported that psychological screening before lumbar surgery revealed preoperative psychological aspects associated with postoperative pain, ADL, and other psychological aspects.

A data mining method using multivariate analysis has the advantage of quantitative expression for clinical interpretation. With this extension, we believe that the patient's more detailed health status can be estimated by natural language analysis (text mining) of the contents of the chart itself. It is also easy to compare with standard physical functions and detect abnormalities. Based on the time series data, we can predict post-surgery conditions and prevent dysfunctions. In a study examining predictive models, Rowena et al. [13] reported that the Orebro–Musculoskeletal Pain Questionnaire of patients with spinal cord-related pain could be used to predict long-term pain and disability. Sinikallio et al. [14] stated that preoperative depression in patients with lumbar spinal canal stenosis affects postoperative physical function, pain, and ADL. The patient’s pain depends on individual pain experiences; also, the pain threshold of residual changes are based on physical, social, and psychological conditions. Pain evaluation is often driven by subjective pain reported by the patient rather than by the affected area's condition. Therefore, a unique objective quantification is required to evaluate pain intensity. We conducted a cross-sectional study that quantitatively predicted the current pain level from a questionnaire rather than a longitudinal prediction to predict future pain levels. For efficient physiotherapy dictated by the patient's degree of pain, physiotherapists must perform an accurate pain assessment of the patient before surgery. Therefore, the patient's pain before surgery can be improved and lead to postoperative ADL and mental improvement [15–17].

**Fig 4:**
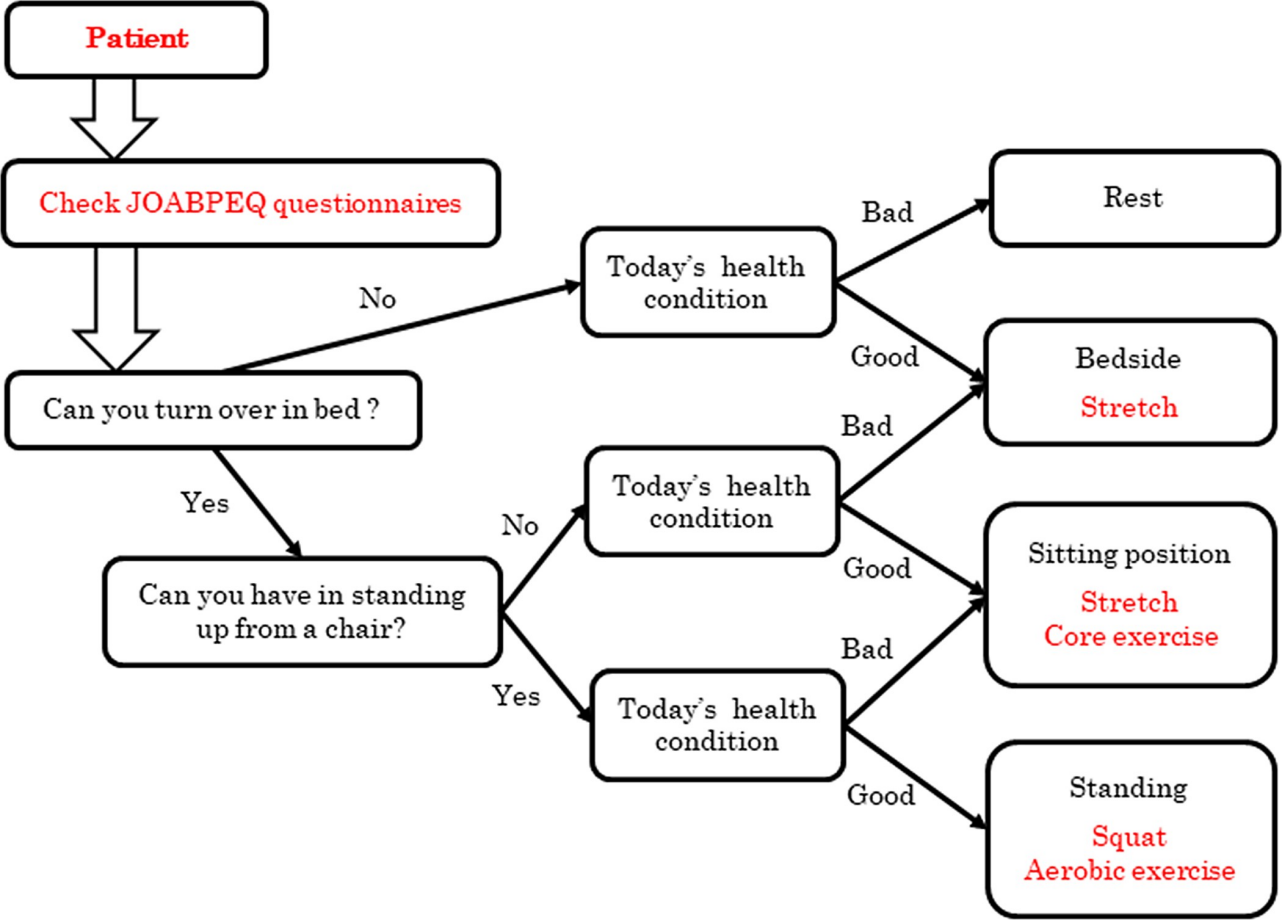
An informative description tree of treatment guidelines for physical therapy in patients with LDH.

As a clinical application, a combination of the subjective VAS, interviews, and high scores of the JOABPEQ for lower back pain and lower limb pain in LDH patients can predict the degree of lower back pain and lower limb symptoms. Specifically, it is necessary to check whether the patient can turn over in bed or stand up from a chair (Q2-3 and Q2-4), depending on their health condition. Physiotherapists can then suggest treatments based on questionnaires. For example, if LDH patients cannot turn over in bed, bedside physical therapy is recommended. If the patients can turn over in bed but cannot stand up from a chair, physical therapy in the sitting position is recommended. If they can turn over in bed and stand up from a chair, physical therapy in the standing position is recommended. Finally, physicians should consider patient health conditions when implementing physical therapy programs on specific days. We propose an informative description tree of treatment guidelines for physical therapy in patients with LDH (Fig 4). For example, a patient visits the hospital's physiotherapeutic division and answers the JOABPEQ to check physical functions. If the patient can turn over in the bed and stand up from a chair, squat or aerobic exercises are recommended as a rehabilitation program, depending on the health condition. The use of the regression model can quantify the subjective degree of lower back pain and lower limb pain and contribute to physical therapy assessment and treatment.

This study's limitation is that the subjects were cases of LDH scheduled for surgery, and cases that could be treated with conservative therapy were not included. Also, the sub-analysis of disease duration, vertebral body level, and hamstring tension was not performed. As future prospects, we will compare the JOABPEQ scores between patients who chose to undergo LDH surgery and those with LDH who did not eventually undergo surgery. Perhaps patients with LDH who require surgery will have a specific response that correlates more strongly than patients with LDH who do not require surgery.

## Conclusions

In this study, we examined whether a regression model can be constructed from the JOABPEQ questions regarding the degree of low back pain and leg pain. Therefore, the degree of low back pain and leg pain correlated with the response to the JOABPEQ, and a regression model could be used.

As a clinical application of LDH patients, by checking the movement of turning over in bed and getting up from a chair, the degree of low back pain and lower limb symptoms can be quantified, contributing to the evaluation of physical therapy and the planning of treatment programs.

## Supporting information

S1 Data (XLSX)
